# The Impact of Neurotoxin Proteins Trafficked by Primary Cilia and Extracellular Vesicles in Neurodegenerative Diseases

**DOI:** 10.3390/biology14121787

**Published:** 2025-12-15

**Authors:** Riley Danna, Soham Kondle, Orr Amar, Michayla Mabourakh, Gratiana Chen, Wala B. Fadol, Ashraf M. Mohieldin

**Affiliations:** 1 Department of Basic Science, College of Medicine, California Northstate University, Elk Grove, CA 95757, USA; riley.danna12170@cnsu.edu (R.D.); michayla.mabourakh9997@cnsu.edu (M.M.);; 2 Department of Clinical Science, College of Medicine, California Northstate University, Elk Grove, CA 95757, USA; 3 Master Program of Pharmaceutical Science, College of Graduate Studies, California Northstate University, Elk Grove, CA 95757, USA

**Keywords:** neurodegenerative disease, primary cilia, extracellular vesicles, tunneling nanotubes, amyloid-beta

## Abstract

Neurodegenerative diseases (NDDs), including Alzheimer’s Disease (AD), Parkinson’s Disease (PD), and Huntington’s Disease (HD), share pathologic mechanisms including oxidative stress, mitochondrial dysfunction, and protein aggregation. However, they differ in age of onset and clinical progression. Emerging evidence highlights primary cilia (PC) as a key regulator of neuronal aging and the progression of these diseases. Dysfunctional PC may impair key signaling pathways, such as Sonic Hedgehog (Shh) and Wnt, promote oxidative stress, mitochondrial damage, and epigenetic instability. PC may also influence intercellular communication by regulating the biogenesis of exosomes and modulating tunneling nanotube (TNT) formation, both of which propagate toxic proteins between neurons. Mechanistically, the regulation of ciliary length is disrupted in AD, which leads to ciliary dysfunction that interferes with signaling pathways and promotes the aggregation of amyloid-beta. This amyloid-beta is then propagated through TNTs and exosomes, spreading neuronal damage. In PD, the accumulation of alpha-synuclein (α-syn) also impairs cilia function, thereby compromising the cell’s response to oxidative stress. This results in the formation of abnormal TNTs and defective exosome-mediated clearance, ultimately contributing to neurodegeneration. Similarly, the mutant huntingtin protein aggregates within primary cilia in HD, morphologically disrupting them by obstructing intraflagellar transport. Damaged cilia are also associated with increased TNT formation and the exosomal release of toxic proteins, which leads to mitochondrial and epigenetic instability, ultimately promoting neuronal aging. Together, targeting ciliary function and its downstream regulation of TNTs and exosomes may provide a novel approach for slowing or halting disease progression across neurodegenerative diseases.

## Simple Summary

This review explores the role of primary cilia and extracellular vesicles in neurodegenerative diseases, including Alzheimer’s, Parkinson’s, and Huntington’s diseases. We discuss how ciliogenesis can disrupt important signaling pathways, as well as its involvement in the aggregation of neurotoxic proteins and the trafficking and clearance of these proteins. We also highlight the promising and novel diagnostic and therapeutic approaches currently being developed to detect or help slow the progression of these diseases.

## 1. Introduction

Neurodegenerative diseases (NDDs) constitute some of the most debilitating diseases facing aging populations today, due to both severity and significant public health ramifications. Despite extensive research efforts, the incidence of NDDs has steadily increased, particularly in Alzheimer’s Disease (AD), Parkinson’s Disease (PD), and Huntington’s Disease (HD). For AD, the most prevalent NDD, concern extends beyond its rising incidence to include a substantial increase in mortality, which has escalated by approximately 140% between 2000 and 2021 [1]. This trend stands in stark contrast to the decline observed over the same period in other leading causes of death in the United States, including stroke, heart disease, and human immunodeficiency virus (HIV) [2]. While the severity of these diseases stems in part from their rising incidence, their profound impact on daily life, as well as the substantial economic burden they impose, highlights the urgency of understanding their pathogenesis and potential intersections [3]. The pathogenesis of AD, PD, and HD can conceptually be broken down into three phases: (1) creation of an abnormal protein, (2) aggregation or lack of clearance, and (3) propagation. This triphasic framework is also shared by other NDDs, such as amyotrophic lateral sclerosis [4,5]. Thus, while these three phases are not exclusive to AD, PD, and HD, they provide essential context for the two primary reasons these three diseases are emphasized over other NDDs in this review.

**Figure 1:**
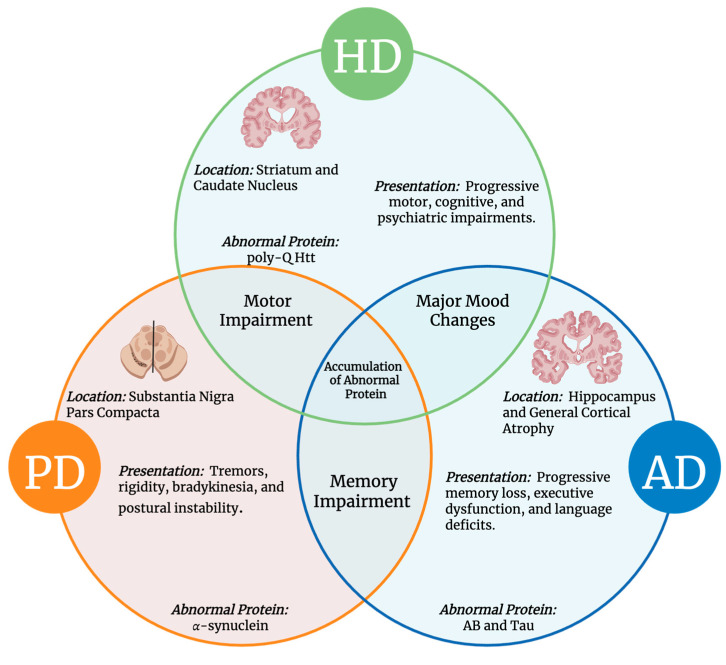
**Common and Unique Features of AD, PD, and HD**. The Venn diagram illustrates overlapping and distinct aspects of these neurodegenerative disorders, including disease location, clinical presentation, hallmark symptoms, and associated neurotoxic proteins [33].

Although different regions of the brain are impacted, there are common and unique characteristics of AD, PD, and HD. While PD and HD both demonstrate motor impairment symptoms that are not typically seen in AD, both AD and PD exhibit memory impairment, which is also observed in HD. However, in these three diseases, the accumulation of neurotoxin proteins is a common feature (Figure 1). In addition, there are distinct linkages between AD, PD, HD, and the dysfunction of primary cilia (PC), as well as two forms of intercellular transport, including tunneling nanotubes (TNTs), and exosomes [6,7,8,9]. PC are microtubule-based, non-motile organelles that extend from the surface of most vertebrate cell types. They act as sensory structures that regulate diverse signaling pathways essential for development and homeostasis [10,11]. This immense structural and functional organelle relies on the coordinated trafficking and temporal localization of specific receptors and their associated signal transduction modules in the cilium [12]. As a center for both sensory and signaling functionality, neuronal pathways like the Sonic Hedgehog (Shh) and Wingless-related integration site (Wnt) are coordinated by PC, allowing for neural tube patterning, synapse formation, neuronal migration, and a host of other functions [13,14,15,16]. To ensure that PC can flexibly conduct the broad array of signaling pathways necessary for proper cellular functioning, cells have evolved diverse mechanisms to monitor and fine-tune cell-type-specific signaling outputs from their cilia in varying developmental and environmental cues [17]. With such a predominant role in cellular signaling, exploring PC’s connection to intercellular transport, and hence communication, is highly warranted. Further, TNTs are thin, F-actin-rich membrane bridges, which are transient and versatile structures that facilitate the bidirectional intercellular transfer of various cargoes, including organelles, plasma membrane components, pathogens, and calcium [18,19]. Exosomes are extracellular vesicles (EVs) derived from endosomes within the cell and whose contents vary depending on their site of origin. Exosomes carry a complex cargo of proteins, lipids, nucleic acids, and other biomolecules that enable them to mediate intercellular communication, modulate signaling pathways, and reflect the molecular signature of their parent cells [20]. Thus, exosomes are produced by almost all cell types [21,22,23,24,25,26]. The functions of exosomes in the brain can be protective or degenerative, depending on the contents and biological signals that they carry [27,28]. This is especially true if the aggregated neurotoxic proteins incur damage to PC, a vital component of neural signaling pathways [29]. Recent proteomic and transcriptomic analyses have revealed disease-specific EV biomarkers across major neurodegenerative disorders. In AD, EVs are enriched in APP fragments, tau, and synaptic proteins; in PD, they carry citrullinated protein signatures, α-syn, SNAP23, and calbindin; and in HD, they contain N-terminal fragments of mutant huntingtin protein with expanded polyglutamine repeats [30,31,32].

Collectively, PC, TNTs, and EVs form an interconnected network of cellular communication that plays a major role in neuronal development. Functionally, PC integrates extracellular cues into intracellular signaling pathways, thereby influencing how cells respond to morphogens and growth factors [9]. TNTs enable rapid and direct exchange of cytoplasmic components such as ions, vesicles, and organelles, providing a structural basis for long-range cell-to-cell transfer [34]. EVs further complement these processes by transporting molecules across the extracellular space, enabling stable intercellular communication and key protein distributions [30,31]. Importantly, emerging evidence suggests that these pathways may not act in isolation; for example, EVs can induce ciliary signaling pathways and ciliogenesis, ciliary signaling can regulate vesicle release, and TNTs have been shown to transfer vesicles between cells [35,36]. Thus, the interplay among PCs, TNTs, and EVs reflects a hierarchical and cooperative system in which localized, direct, and extracellular modes of communication reinforce one another to coordinate complex cellular behaviors.

While the pathways leading to ciliary dysfunction vary across AD, PD, and HD, the downstream effects remain consistent, where impaired primary cilia amplify aging-related damage through three key processes: (1) oxidative stress, (2) mitochondrial dysfunction, and (3) epigenetic alterations [34,35]. However, there is currently a gap in the literature that explores the interplay between PC, TNTs, and EVs in the context of these three diseases. Therefore, in this review, we examine the role of PC as a key regulator of neuronal development and their involvement in intercellular communication via TNTs and EVs. Additionally, this review will discuss both the common and unique elements of epidemiology, pathogenesis, preventive measures, diagnostic strategies, and therapeutic applications related to these three diseases.

## 2. Methods

For this literature review, a comprehensive search of existing studies on TNTs, exosomes, and primary cilia in neurodegenerative diseases was conducted using the academic databases PubMed and Google Scholar. The search terms included “tunneling nanotubes,” “exosomes,” “primary cilia,” “neurodegenerative diseases,” “Alzheimer’s disease,” “Huntington’s disease,” “Parkinson’s disease,” “ciliopathy,” and “cilia.”

Literature was screened for studies that addressed the intersection of TNTs, exosomes, and primary cilia in the context of neurodegenerative diseases. Exclusion criteria included all other neurodegenerative diseases, such as “Frontotemporal Dementia”, “Amyotrophic Lateral Sclerosis”, and “Lewy Body Dementia”, among others.

Both original research articles and review papers were included to capture important findings and emerging perspectives. Additional studies identified through the reference lists of selected papers were included when relevant. Emphasis was placed on literature addressing protein aggregation, cellular communication, and alterations in ciliary signaling pathways relevant to disease progression.

## 3. Epidemiology and Clinical Presentations

**Figure 2:**
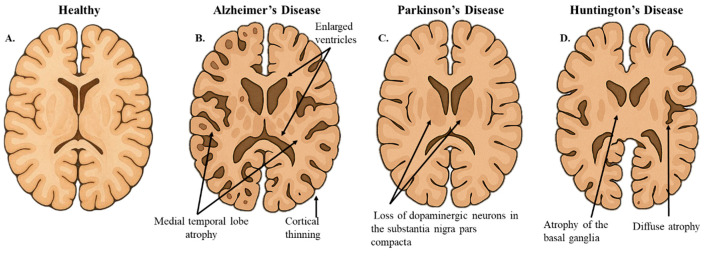
**Hallmarks of Neurodegenerative Brain Morphology**. (**A**). The illustrative image represents a coronal section of healthy brain morphology. (**B**). In AD, the coronal slice shows cortical thinning and medial temporal lobe atrophy. (**C**). In PD, the coronal slice highlights the pallor of the substantia nigra, indicative of dopaminergic neuron loss. (**D**). In HD, the coronal slice demonstrates diffuse cerebral atrophy and atrophy of the basal ganglia. This figure was created using OpenAI GTP-4o.

AD has emerged as one of the leading causes of physical and cognitive disability globally, accounting for almost 80% of all reported dementia diagnoses [36,37,38,39]. With its rising prevalence, AD is quickly becoming one of the most costly and lethal neurodegenerative diseases, placing a significant burden on healthcare systems and long-term care facilities. AD is marked by a slow decline in cognitive abilities, including memory, executive function, and language skills [40,41]. Early in AD progression, damage to neuronal connections occurs primarily in the medial temporal lobe and neocortical areas (Figure 2B) [42,43].

PD, the second most common neurodegenerative disease, follows a similar pattern to AD, albeit on a smaller scale [44]. A global study analyzing trends in PD reports that the incidence of the disease has increased by an average of 61% per year from 1990 to 2019 [45]. PD is associated with four cardinal motor symptoms, including tremors, rigidity, bradykinesia, and postural instability, alongside the characteristic shuffling and stooped posture of the “Parkinsonian gait” [46]. Progressive neurodegeneration is most pronounced within the nigrostriatal dopaminergic neurons of the substantia nigra pars compacta, a region of the brain associated with movement control and reward behaviors (Figure 2C) [47].

HD, in contrast to AD and PD, is a rarer but highly severe neurodegenerative disorder with a global prevalence of 0.48 per 100,000 person-years (2010–2022), and a U.S. prevalence of 1.76 per 100,000 person-years in the same period [48]. Unlike AD and PD, HD follows an autosomal dominant inheritance pattern and presents earlier in life, typically between 30 and 50 years [49,50]. HD is primarily characterized by progressive motor, cognitive, and psychiatric impairments [51]. Markedly, the frontostriatal circuits are among the earliest and most consistently affected brain regions in HD, followed by more widespread atrophy of the frontal and temporal regions, and eventually deeper subcortical structures, including the limbic system and cerebellum (Figure 2D) [52,53].

Emerging evidence from both mouse models and post-mortem analyses of human brains indicates changes in the morphology of PC, with several studies reporting differences in ciliary length and abundance [54,55]. While these alterations are consistently observed in NDDs, the relationship between clinical manifestations and ciliogenesis remains correlational rather than causative.

## 4. Mechanisms and Etiology

Aging is widely regarded as the most significant risk factor influencing the NDDs’ progression, severity, and impact [56]. This is due to the brain’s limited regenerative capacity compared to tissues, such as skeletal muscle and skin, which rely on stem cells to repair damage [57,58]. When neurons and oligodendrocytes sustain age-related damage through altered DNA methylation, mitochondrial dysfunction, and reactive oxygen species (ROS), their lack of regenerative capacity renders them more susceptible to irreversible damage compared to other cell types [59,60]. In AD, PD, and HD, aging has also been associated with increased telomere attrition [61,62,63]. Consequently, the absence of telomerase activity, coupled with poor regenerative capacity, creates an environment that is conducive to the progression of neurodegenerative disease. Despite their shared pathological stages, these diseases display striking differences in the average age of onset [64,65,66,67]. As discussed later in this review, age-related cellular stressors not only heighten neuronal vulnerability but also alter PC function and modulate EV and TNT-mediated propagation of pathogenic proteins, further shaping NDDs progression.

### 4.1. Alzheimer’s Disease Pathogenesis

AD is a multifactorial, progressive neurodegenerative disorder primarily affecting the brain’s memory and cognitive functions. Pathogenesis of AD has been linked to genetic alterations and the accumulation of two proteinaceous lesions in the brain, amyloid-beta plaques (Aβ) and abnormally phosphorylated tau tangles, that disrupt neural communication and impair synaptic transmission. These changes interrupt neuronal processes and extend to their larger networks, impeding communication, metabolism, and repair [68]. Together, Aβ plaques and tau tangles are referred to as the amyloid tau neurodegeneration (ATN) biomarkers [69]. Genetic mutations associated with AD, such as those in the APP gene on chromosome 21, PSEN1 and PSEN2 on chromosomes 14 and 1, respectively, result in altered processing of APP and excessive production of Aβ, which aggregates into plaques [70]. These plaques, in turn, are thought to trigger neuroinflammation and neuronal toxicity, initiating a cascade of events that lead to neuronal death [71]. The other proteinaceous lesion associated with AD, Tau, a protein that normally stabilizes microtubules, is critical to guiding nutrients and molecules from the cell body to the axon and dendrites [72]. In AD, tau becomes hyperphosphorylated, causing it to detach from microtubules and form neurofibrillary tangles with other tau molecules [73]. These tangles disrupt axonal transport, leading to a loss of synapses and further neuronal death [74]. As neuronal damage increases, neuronal networks break down, and susceptible areas of the brain begin to atrophy [75]. The resulting neurodegeneration predominantly affects the hippocampus, the brain region critical for memory formation, contributing to the hallmark cognitive decline seen in AD patients [76,77]. Another study has also linked impaired neurogenesis to AD-related memory and cognitive deficits [78]. As the disease progresses, atrophy spreads to the temporal, parietal, and frontal lobes of the brain, leading to widespread cortical dysfunction [79,80].

#### Primary Cilia, Exosomes, and TNTs in Alzheimer’s Disease

Degeneration of ciliary signaling pathways reduces cell proliferation and contributes to age-related diseases, such as AD [81]. Microglia, the brain’s phagocytic cells, maintain homeostasis by responding to injury, pruning synapses, and promoting tissue repair [82,83]. In AD mouse models, both the number and morphology of microglial PC are significantly reduced [84,85,86]. Disease-associated microglia (DAM) actively phagocytose dystrophic neurites and Aβ plaques [87,88]. Notably, inhibition of primary cilia in septal microglia leads to larger Aβ plaque accumulation and increased neurite damage [84]. Further, APP has been demonstrated to localize to the PC along with the Shh signaling component Smo [89]. In addition, brain regions with greater Aβ deposition fail to show the normal age-dependent increase in neuronal cilia length [90]. Interestingly, a study found that dentate granule cell primary cilia were shortened by ~50% only in models expressing both Aβ and tau, whereas models expressing Aβ alone or tau alone showed no significant change [9]. Additionally, the most supported mechanism for Aβ-induced cilia shortening involves structural distortion and disruption of Shh signaling, which is essential for ciliary structure and elongation [91]. Moreover, the alterations in cilia length and frequency correlate with Aβ plaque burden and disease severity [91]. AD pathogenesis begins with APP being cleaved abnormally by a β or ɣ-secretase, producing Aβ, which later aggregates to form plaques [92,93,94,95]. Tau, a microtubule-associated protein, subsequently becomes hyperphosphorylated and forms intracellular tangles [73,96]. APP and other AD-related proteins accumulate at the ciliary basal body, contributing to shortening of cilia and subsequent dysfunction of ciliary signaling and neuroprotective pathways [29,89,97]. Ultimately, Aβ and tau propagate to other neurons via TNT and exosomes [98,99,100,101].

**Figure 3:**
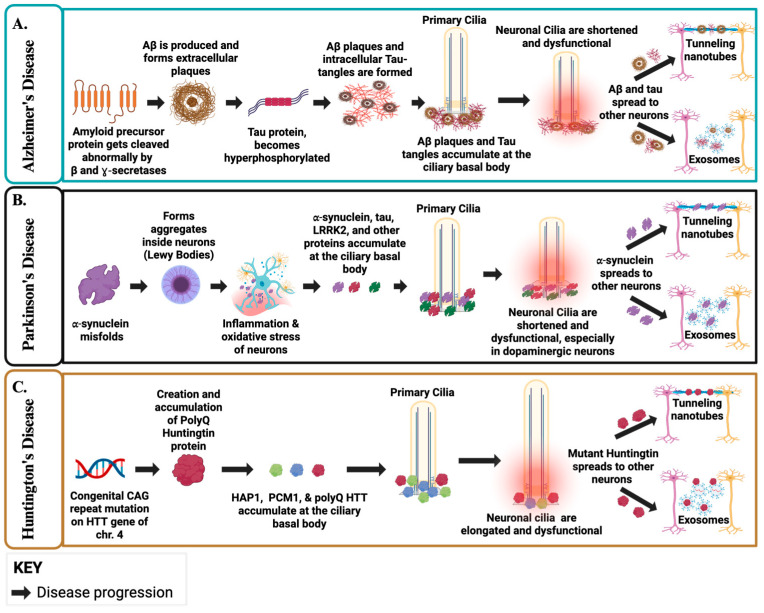
**Ciliary Dysfunction and Protein Propagation in AD, PD, and HD**. (**A**). In AD, Abnormal cleavage of APP generates Aβ, which accumulates at the ciliary basal body, leading to shortened and dysfunctional cilia and impaired signaling. Hyperphosphorylated tau forms tangles, and both Aβ and tau propagate via EVs and TNTs, beginning in the neocortex and entorhinal cortex before spreading more broadly. Disrupted ciliary–autophagy coupling diverts pathogenic cargo toward EV release, amplifying intercellular propagation of toxic proteins. (**B**). In PD, misfolded α-syn aggregates into Lewy bodies at the basal body, damaging cilia in dopaminergic neurons and impairing Shh signaling. Loss of primary cilia and impaired autophagy enhance EV-mediated α-syn export, while lysosomal damage further drives cargo release. TNT formation, promoted by actin remodeling under ciliary control, provides an additional pathway for α-syn spread from brainstem nuclei upward into cortical circuits. (**C**). In HD, mutant huntingtin (HTT) accumulates at the centrosome/basal body, producing elongated and dysfunctional cilia and disrupting cilia-dependent signaling. Defects in autophagy and lysosomal trafficking redirect cargo toward secretory pathways, increasing EV release of HTT. Ciliary signaling also directly regulates actin-dependent EV biogenesis. In parallel, actin remodeling promotes TNT formation, including Rhes-driven “tunnels” that transport HTT between neurons, complementing EV-mediated spread observed in patient biofluids. Created in BioRender. Danna, R. (2025) https://BioRender.com/jasqo6c [189].

Furthermore, PC are key regulators of autophagy-endolysosomal pathways, and when ciliary-autophagic trafficking is disturbed, ciliary cargo can be redirected toward EV secretion [102,103,104]. These pathway alterations enhance the EV-mediated export of pathogenic proteins, including Aβ and tau, facilitating their propagation between cells [98,99]. Concurrently, AD-related cellular stressors, such as Aβ exposure, activate actin remodeling pathways that may drive TNT formation, enabling direct neuron-to-neuron transfer of pathological tau protein [100,101]. These coordinated defects in ciliary signaling and intracellular trafficking may collectively facilitate the EV and TNT-dependent propagation of neurotoxic proteins across neural networks, thereby potentially reinforcing progressive circuit dysfunction (Figure 3A) [98,100,101,105].

Chronic inflammation in the brain may be associated with the buildup of malfunctioning glial cells, which are normally responsible for clearing debris [83,106]. Studies in both animal and human models show that increased Aβ plaque burden correlates with dysregulation of key ciliary signaling pathways, including Shh and Wnt [107,108,109,110]. The Shh pathway displays upregulation of its components, Shh, SMO, GLI1, and GLI2, in the hippocampus of human and mouse AD models, whereas PTCH, GLI3, and Wnt signaling components are downregulated [111,112]. Similar to other neurodegenerative diseases, dysfunction of these cilia-dependent pathways in AD has been associated with disturbances in critical homeostatic processes governed by PC [113,114].

In physiological conditions, exosomes assist neuronal function and intercellular communication, serving as transporters for lipids, proteins, miRNA, and mRNA [115]. However, in damaged environments, like AD, exosomes are involved in the transportation of damaging proteins like Aβ and Tau [116]. Further, an intracranial injection of exosomes from AD mice brains into WT mice caused marked mitochondrial damage and neurotoxicity [117]. The exosomes isolated from the AD mice showed significantly higher levels of tau and Aβ 1–42 proteins [117]. A recent article introduced TNTs as potential contributors to the pathogenesis of AD [118]. As mentioned above, TNTs may play a significant role in the spreading of misfolded proteins like Aβ and Tau, which are central to AD pathology [100]. TNTs allow the direct transfer of these pathogenic aggregates between neurons, bypassing the extracellular space where they might be cleared by immune cells [101,119]. This facilitates the propagation of AD pathology in a predictable, spatiotemporal manner, supporting the progression of the disease. Moreover, TNT formation is often associated with oxidative stress, another key feature of AD [120,121,122]. Given their role in intercellular communication and the spread of protein aggregates, TNTs are emerging as potential therapeutic targets for slowing the disease’s progression [100,123,124].

### 4.2. Parkinson’s Disease Pathogenesis

The etiology of PD is currently thought to be multifactorial. However, the exact mechanism underlying Parkinsonian symptoms, including bradykinesia, tremor, and postural instability, remains largely unclear. The current understanding of PD pathogenesis suggests that a combination of genetic and environmental factors contributes to the progressive neurodegeneration of nigrostriatal dopaminergic neurons in the pars compacta of the substantia nigra, as well as other affected cortical regions [125,126]. This lack of dopamine is understood to be the primary cause of many of the movement disorders [127,128].

One of the main mediators of this neurodegeneration is the α-syn protein. Derived from 140 amino acids, α-syn is made of three main groups: a highly conserved N-terminal, a hydrophobic non-amyloid β compound, and an unstructured C-terminus [129]. Similar to Aβ and Tau protein in AD, α-syn causes extensive damage when it becomes misfolded [130]. In PD, aggregation of α-syn leads to the formation of Lewy Bodies, a notable characteristic marker of the disease [131]. The aggregation of abnormal Lewy Bodies causes resulting damage and neuroinflammation, leading to deficits and disruption in neurotransmitter signaling [132]. Notably, although α-syn appears early in peripheral tissues, brain damage in PD begins in the brainstem and spreads to the cortex [125,133,134,135]. While the misfolding of α-syn protein is considered to be the main issue in PD, its impact is further exacerbated when it is propagated and not cleared properly [136].

#### 4.2.1. Genetic Relevance in Parkinson’s Disease

Despite most cases of PD resulting from sporadic mutations, inherited PD remains a legitimate concern and is highly correlated with many genes, including Synuclein alpha (SNCA), Leucine-rich Repeat Kinase 2 (LRRK2), Parkin (PRKN), PTEN Induced Kinase 1 (PINK1), VPS35 Retromer Complex Component (VPS35), and Vacuolar Protein Sorting 13 Homolog C (VPS13C) [137]. While α-syn does the most damage, mutation of the gene responsible for its production, the SNCA gene, does not have sole responsibility for the manifestation of PD. In fact, no single gene has complete penetrance in PD, but rather, different combinations of genes have been shown to have an effect in causing PD [138]. Given the three-pronged approach to pathology that α-syn has, this stands to bear reason.

Beyond SNCA mutations, another key gene implicated in PD pathogenesis is PINK1, which encodes a protective mitochondrial Serine/Threonine kinase [139]. Collectively, mutations in these two genes illustrate dual problems in PD pathogenesis: SNCA mutations disrupt protein formation and PNK1 mutations disrupt clearance of these misfolded proteins. Normally, PINK1 serves a protective role by helping to produce the PTEN-induced kinase protein, which shields mitochondria from damage caused by the reactive oxygen species (ROS) [140]. In PD, where there is an overexpression of α-syn, there is an increase in ROS generation, and PINK1 ameliorates the resulting damage. However, when PINK1 is mutated, it loses its function, which means there is no longer any protection against ROS [141]. Thus, this lack of PINK1 leads to the accumulation of α-syn, which further damages the mitochondria [142].

#### 4.2.2. Primary Cilia, Exosomes, and TNTs in Parkinson’s Disease

While α-syn’s role in causing physical damage is a pivotal piece of PD, α-syn has also been shown to further this neurological ailment through faults in cellular signaling and autophagy due to ciliary dysfunction [143]. Emerging evidence has shown that the LRRK2 gene is associated with both α-syn accumulation and PC [144]. The mutation of LRRK2 leads to the hyperactivation of Rab-GTPases, which have been directly shown to decrease ciliogenesis [145].

Furthermore, α-syn aggregates to form complexes called aggresomes that localize adjacent to the centrosome, or the basal body of the cilium, which inhibit ciliary function and ciliogenesis [143]. Next, the loss of cilia function in PD, driven by pathogenic LRRK2 accumulation, disrupts Shh signaling, causing dopaminergic neurons to lose vital growth and survival support, thereby increasing their vulnerability to degeneration [146,147,148]. Similar to AD, disruption of cilia–autophagy crosstalk in PD has been shown to cause neurons to redirect α-syn into EVs, increasing its intercellular transfer, while autophagy inhibition further amplifies α-syn-containing EV release and spread [149,150]. Concurrently, α-syn fibrils damage lysosomes and propagate between neurons via lysosome-associated cargo transported through TNTs, providing a TNT-dependent route for pathology spread [151,152]. Because TNT formation relies on actin-dependent machinery (e.g., M-Sec/RalA/exocyst) and ciliary Shh signaling regulates neuronal actin dynamics, ciliary dysfunction may promote TNT formation, while impaired autophagy increases EV loading, collectively enhancing α-syn dissemination (Figure 3B) [102,149,150,153,154,155].

Currently, there are two main proposed mechanisms for neurodegenerative spread. First, TNTs have long been postulated to play a role in both communication and transportation between distant cells [156]. A recent study has shown that TNTs can indeed transfer cellular components between cells [157]. In PD, a primary cell can get overloaded with α-syn and then use TNTs to dispose of the excess protein, which then gets spread to other cells [118]. Second, the propagation of α-syn has been proposed to be trafficked by EVs. Notably, vesicles transfer a diverse cargo of cellular materials, fuse with the plasma membrane, and are then released to target other cells [156]. While TNTs represent a more localized and direct transfer of α-syn, exosomes have been shown to spread contents of one cell, such as organelles, plasma membrane components, pathogens, and calcium, to another across far distances [20]. Microglia-derived exosomes have also been shown to carry and transfer pathogenic α-syn species, potentially further contributing to this spread [158]. Moreover, strain-specific α-syn conformations exhibit distinct propensities for exosomal packaging, likely driving variability in “prion-like” propagation [159]. However, the exosomes implicated in PD pathogenesis are not limited to transferring only α-syn. Emerging evidence indicates that EVs may also transfer faulty genetic material, such as miR-1, miR-19b, miR-153, and miR-409-3p, to other cells, suggesting an additional mechanism for disease propagation [160].

### 4.3. Huntington’s Disease Pathogenesis

Huntington’s Disease arises from an expansion of cytosine-adenine-guanine (CAG) trinucleotide repeats in the HTT gene (formerly IT15) of chromosome 4p16.3, leading to the production of a dysfunctional huntingtin protein (polyQ-HTT) [161,162]. The unstable CAG repeats in exon 1 of HTT encode the N-terminal polyglutamine tract of the ~350 kDa, ~3100 amino acid huntingtin protein [163]. The normal huntingtin protein is multifunctional, with roles in transcriptional regulation, vesicular transport, and synaptic transmission [164,165,166]. Elongation of polyglutamine stretches in the abnormal protein is currently regarded as the causal factor for normal huntingtin’s pathogenic transformation, specifically by way of losing its normal solubility in nerve cells and aggregating into intraneuronal inclusions in different compartments of vulnerable brain regions [167,168].

Similar to α-syn in PD and Tau in AD, polyQ-HTT aggregates exert toxic gain-of-function effects, forming β-sheet-rich structures that disrupt essential cellular pathways and cause neurotoxicity [169,170]. Impaired intracellular transport, a hallmark of HD, is closely linked to PC dysfunction, which can alter ciliary structure and disrupt the trafficking of signaling molecules regulating Shh and Wnt pathways [54,171,172]. Further, in HD models, ciliary elongation has been linked to disrupted intraflagellar transport (IFT), a process essential for trafficking signaling molecules along the ciliary axoneme [173]. While these findings establish a connection between ciliary alterations and impaired trafficking, the underlying mechanisms remain poorly understood [174]. Moreover, while both ciliary elongation and IFT disruption have been reported in HD models, supporting evidence from human post-mortem tissue remains limited [69,175]. In contrast to AD and PD, where ciliary involvement is increasingly recognized, the role of cilia in HD pathogenesis remains underexplored [176,177,178].

#### Primary Cilia, Exosomes, and TNTs in Huntington’s Disease

Pathogenic polyQ-HTT promotes the accumulation of huntingtin-associated protein 1 (HAP1) and pericentriolar material 1 (PCM1) at the centrosome, leading to elongation of PC and accumulation of N-acetylated tubulin in HD neurons [54,179]. Normally, huntingtin transits between the basal body and cilium via phosphorylation within its N17 domain, but polyQ-HTT exhibits reduced N17 phosphorylation, resulting in accumulation along the ciliary stalk [180,181].

Defective autophagy and lysosomal trafficking in HD further redirect neuronal cargo to secretory pathways, enhancing exosome release and facilitating mutant HTT export through late endosomal and lysosomal routes [182,183]. Ciliary signaling regulates actin networks and drives ciliary exocytosis, linking cilium function to EV biogenesis [184,185]. In HD, actin remodeling promotes TNT formation, with Rhes-driven “Rhes tunnels” and TNTs facilitating intercellular transfer of mutant huntingtin and polyglutamine aggregates, potentially complementing EV-mediated export observed in patient biofluids (Figure 3C) [186,187,188].

Beyond aggregation, polyQ-HTT impairs protein clearance pathways, exacerbating cellular dysfunction, and disrupts autophagy by impairing lysosomal degradation and cargo recognition [190]. New emerging evidence shows that mutant HTT sequesters key autophagy regulators, such as p62 and LC3, preventing the clearance of damaged organelles and misfolded proteins [191]. This defect leads to further accumulation of mutant HTT and other toxic components, exacerbating neuronal stress and accelerating death [54]. In addition to direct neuronal toxicity, HD pathogenesis is accompanied by significant neuroinflammation [192]. Brains impacted with HD exhibit increased microglial activation and elevated pro-inflammatory cytokine levels, including IL-6 and TNF-α [193,194]. Further, mutant HTT has been shown to trigger an elevated immune response, leading to chronic neuroinflammation that increases neuronal damage [171,195].

In addition to disrupted intracellular transport, accumulating evidence suggests that mutant HTT propagates between neurons via TNTs and EVs, including exosomes carrying misfolded protein cargo [186,187,196]. Notably, exosomes derived from HD patient neurons contain mutant HTT, providing direct evidence that exosome-mediated transfer contributes to disease spread [197].

## 5. Preventive Measures, Diagnostics, and Therapeutic Strategies

NDDs remain a major cause of disability and mortality worldwide, with no definitive cure. Increasing evidence suggests that a combination of lifestyle modifications, diagnostic innovations, and emerging therapeutic strategies can significantly influence disease onset, progression, and management. Of particular interest are EVs, especially exosomes, which have emerged as critical mediators of intercellular communication, pathogenic protein propagation, and potential therapeutic delivery. The following section highlights recent advances in preventive strategies, diagnostic modalities, and therapeutic approaches across AD, PD, and HD.

### 5.1. Preventive Measures

There have been many strategies to delay or prevent the progression of NDDs, including lifestyle changes, physical activities, and high cognitive reserve. For instance, maintaining a healthy lifestyle through balanced caloric intake and adequate sleep supports the structural and functional integrity of PC [198,199,200,201]. In return, this may enhance the activation of ciliary EVs (ciEVs) and neuroprotective signaling pathways like Shh and Wnt, contributing to neuronal resilience and potentially reducing the risk of NDDs. Congruently, recent studies have increasingly focused on lifestyle factors as the mainstay of preventive strategy against the development and progression of NDDs [202,203]. In AD, poor sleep quality and shorter sleep duration are significantly correlated with higher Aβ levels in non-demented populations [204]. Adequate amounts of sleep per night, around 7 h or more, powerfully attenuates oxidative stress and neuroinflammation, key drivers of neurodegeneration, lowering the risk of neurodegenerative disease [205].

While the direct impact of physical activity on TNT formation remains unclear, maintaining neuronal health through exercise may influence TNT dynamics. However, regular exercise has been shown to enhance PC length and ciliogenesis in astrocytes, promoting neuroprotective effects and improving cognitive function in animal models of chronic cerebral hypoperfusion [206]. In addition, physical activity has also been shown to induce the release of EVs carrying neuroprotective factors, which may contribute to the beneficial effects of exercise in preventing NDDs [207]. In particular, higher levels of physical activity have been shown to reduce the risk of AD and PD [208]. In HD, exercise training stabilized patients’ motor deficits and increased their peak oxygen uptake and cardiovascular function [209].

Notably, NDDs, including AD, PD, and HD, tend to exhibit accelerated disease progression and worsening symptoms under stress [210,211,212]. Consistently, it has been shown that stress may significantly influence PC, EVs, and TNTs, modulating neuronal signaling and intercellular communication. In return, PC may alter neuronal excitability and sensitivity, EVs may modify their cargo and release, transmitting stress signals that influence neuroinflammation and synaptic plasticity, and TNTs may increase under oxidative or cellular stress, potentially propagating both protective and pathological effects [213,214,215]. To attenuate this effect, both yoga and mindfulness meditation have been shown to improve health-related quality of life by reducing anxiety and depressive symptoms in neurodegenerative diseases [216,217].

#### 5.1.1. Diagnostics in Alzheimer’s Disease

AD can be confirmed only through a postmortem brain autopsy [218]. In living patients, diagnosis is based on cognitive assessments and cerebral spinal fluid (CSF) biomarkers, such as Aβ, total tau, and phospho-tau-181, using enzyme-linked immunosorbent assay (ELISA) analysis [219,220]. Since CSF collection is invasive, positron emission tomography (PET) and magnetic resonance imaging (MRI) scans are used as noninvasive methods to detect metabolic and structural brain changes associated with AD [221,222,223]. However, alterations in neuronal PC and TNTs may also serve as early mechanistic indicators, but no clinical tools currently exist to evaluate or quantify them in patients. Instead, ongoing clinical trial studies are investigating EVs’ cargo, such as Aβ and tau proteins, for potential AD markers, due to their superior sensitivity, specificity, and accessibility in saliva, blood, and other body fluids [224,225].

Currently, there are two clinical trials aimed at diagnosing AD using EV biomarkers. One actively recruiting clinical trial (NCT06869135) is investigating the potential utilization of Raman Spectroscopy to identify biomarkers of neuronal damage and inflammation present in saliva and salivary extracellular vesicles (sEVs), as a novel approach to early diagnosis of NDDs. By analyzing and tracking the progression of salivary components, focusing on the contents of sEVs, the study aims to facilitate primary early diagnosis, accurately monitor stages of progression, as well as isolate associated biomolecular markers. The other clinical trial (NCT03381482) aims to measure tau quantity in EVs from cerebrospinal fluid (CSF), lumbar puncture, or blood collected from patients at varying AD risk and stages. Collectively, in clinical diagnostic and prognostic studies of AD, EVs have been isolated from plasma, CSF, blood, and saliva, originating from stem cells, neurons, and immune cells. If successful, some of these clinical trials could serve as a noninvasive and early diagnostic tool for AD.

#### 5.1.2. Therapeutics in Alzheimer’s Disease

Currently, there are no clinical studies or approved AD therapies that specifically target PC or TNT. However, mesenchymal stem-cell (MSC)-derived exosomes have been shown to reduce Aβ expression and restore the expression of neuronal memory and synaptic plasticity-related genes in a cell culture model of AD [226,227]. Furthermore, MSC-derived exosomes have been shown to significantly improve brain glucose metabolism and cognitive function compared to controls in AD mice [228]. Neural-stem cell (NSC)-derived exosomes have been shown to alleviate mitochondrial dysfunction, enhance synaptic activity, and decrease inflammation in mice [229]. NSC exosomes have also been shown to effectively repair damage to the blood–brain barrier induced by Aβ [230]. Despite their strong therapeutic promise in AD, the production of stem cell-derived exosomes remains challenging. Their yield from cultured media is extremely low, posing a major challenge for large-scale manufacturing and clinical translation [231]. As a result, strategies such as 3D culture systems, bioreactors, or chemical stimulation are being explored to enhance exosome yield and scalability for therapeutic applications [232,233,234].

Traditionally, AD treatment relied on cholinesterase inhibitors and memantine to manage symptoms [235,236,237]. At present, one clinical trial is assessing MSC-derived EVs as a potential therapy for AD, utilizing intranasal administration of EV-containing drops (HUC-MSC-sEV-00; NCT0660790). The trial is currently in the recruiting phase and will assess therapeutic outcomes using neuropsychological evaluations, without molecular assessment of pharmacological effects.

#### 5.1.3. Diagnostics in Parkinson’s Disease

Currently, PD is primarily diagnosed through the presence of clinical symptoms. Due to PD having multiple variants of disease, accurate and thorough history taking allows clinicians the best opportunity to make the proper diagnosis [238]. Understanding variant differences helps clinicians understand more accurate prognoses, as some variants are more aggressive than others [239]. Typically, bradykinesia, a symptom of a more dominant variant, occurs in concert with either tremor or rigidity, or both [239]. To prevent misdiagnoses in situations where Parkinsonian symptoms are present, but the disease course is aggressive or at an atypical age, dopamine transporter single-photon emission computed tomography is used to confirm PD [240].

Similar to AD, EVs are being actively investigated in clinical trials for the diagnosis and potential treatment of PD. EVs isolated from saliva, blood, urine, and cerebrospinal fluid are evaluated for multiple PD-associated markers. Notably, a recently developed assay identified membrane-associated α-syn in EVs as an early-stage biomarker for PD [241]. Although these assays are not yet standard clinical practice, there are ongoing studies aiming to validate their clinical utility. Currently, different clinical trials are examining EVs isolated from biofluids such as urine, blood, CSF, and saliva for PD diagnostic applications. Of particular interest, several trials have focused on LRRK2-derived EVs (NCT01860118, NCT04603326, NCT03775447) as potential PD biomarkers, which are known to regulate vesicle trafficking and cytoskeletal dynamics associated with PC and TNTs.

#### 5.1.4. Therapeutics in Parkinson’s Disease

Current PD treatments primarily use a combination of carbidopa and levodopa medications to improve motor symptoms [242]. However, side effects, such as dyskinesias, can be managed with dopamine agonists or monoamine-oxidase-B inhibitors, with deep-brain stimulation serving as an option for patients unresponsive to medications [243]. In contrast, EVs may cross the blood–brain barrier and can deliver proteins, RNAs, or drugs directly to target cells, enabling disease-modifying, cell-specific, and multifunctional therapy beyond symptomatic relief [244]. As a result, EVs have been used to transfer PD-specific drugs, specifically targeting dopaminergic neurons [245]. In addition, genetic material such as miR-124-3p can also be transported, which has shown promise in preventing dopaminergic neuron degradation [246]. Similar to AD, the introduction of NSCs has also been shown to increase neuroprotection, especially when enriched with catalase, which is an antioxidant protein [247]. Further, there are currently no clinical studies that specifically target PC or TNT for PD therapy. Instead, a couple of clinical trials (NCT06607900, NCT05152394) are currently evaluating intranasal-derived EVs from human umbilical cord mesenchymal stem cells (hUC-MSCs) as a therapeutic application for PD. The goals for these trials are to modulate neuroinflammation, support neuronal survival, and target disease pathways by delivering bioactive proteins and RNAs directly to the central nervous system (CNS). The trials primarily assess safety, tolerability, and preliminary efficacy using motor and cognitive function tests.

#### 5.1.5. Diagnostics in Huntington’s Disease

HD is usually confirmed diagnostically through the identification of 36 or more CAG repeats on the short arm of chromosome 4p16.3 in the Huntingtin gene [162]. The 36–39 repeat range leads to incomplete penetrance or very late onset of the disease, while definite clinical manifestation is seen at repeat levels of above 40 [248]. One of the most pertinent genetic phenomena in HD is called anticipation, which is when the number of repeats exceeds 55, increasing in each generation [249]. This process can result in Juvenile HD, an aggressive form of the disease that has a significantly earlier onset [250]. To both monitor and predict HD’s manifestation in individuals and across generations, three main categories are assessed: (1) imaging, (2) CSF biomarkers, and (3) bloodborne biomarkers [251,252]. Key biomarkers include polyQ-Htt and Tau, which are detected in the CSF, as well as BDNF, which is measurable in the blood [253,254,255,256]. Imaging is often magnetic resonance imaging, which is useful in understanding cerebral volumes, blood flow, and structural changes [257].

While PC and TNTs have been implicated in HD pathophysiology, they are not yet validated as robust diagnostic tools. Currently, only one clinical trial (NCT06082713) is investigating the HD marker HTT in blood-derived EVs. Moreover, platelet-derived EVs from both pre-manifest and manifest HD patients were shown to carry mutant huntingtin protein; however, EV release levels did not significantly differ from controls, limiting their present diagnostic utility [258].

#### 5.1.6. Therapeutics in Huntington’s Disease

In HD, current therapies revolve around the treatment of symptoms, such as the use of tetrabenazine to treat chorea, involuntary and irregular movements, one of the hallmark symptoms [259]. As for exosomes or TNTs, there is little evidence of these being useful in actual symptom reduction. However, similar to PD, exosomes containing miRNA have shown promise, as their uptake in the brain has been observed in patients with HD [260]. In fact, miR-124, a miRNA shown to be repressed in HD, was utilized in a mouse study via an exosome-based delivery method. While miR-124 delivery was not successful in behavioral symptom reduction, it still demonstrated promise. This is because it could serve as a vehicle for delivering a different miRNA within the exosomes that might have a therapeutic effect. However, there is no current clinical trial investigating either PC, EVs, or TNTs as a potential therapeutic application. For recent clinical trials investigating the diagnostic, prognostic, and therapeutic applications of EVs in AD, PD, and HD, refer to Table 1.

## 6. Discussion

The increasing prevalence of NDDs underscores the urgent need for improved diagnostics, therapeutics, and care strategies. Early detection combined with emerging therapeutics could slow disease progression and improve quality of life. The shared mechanisms across AD, PD, and HD highlight the critical roles of PC, TNTs, and EVs in NDDs. While each disease has unique etiological factors, the convergence of ciliary dysfunction and protein propagation emphasizes the importance of studying these components in tandem.

PC are essential organelles in neurodevelopment, acting as hubs for major signaling pathways, which regulate brain structure formation and cellular differentiation [259,260,261,262]. During early embryonic development, the disruption of these cilia-associated signaling pathways can result in neurodevelopmental abnormalities [261]. Functionally, these pathways are vital for the proper formation and spatial organization of the brain [262]. Moreover, these signaling pathways also govern cell fate decisions, influencing processes such as differentiation, survival, and programmed cell death [114,263].

**Figure 4:**
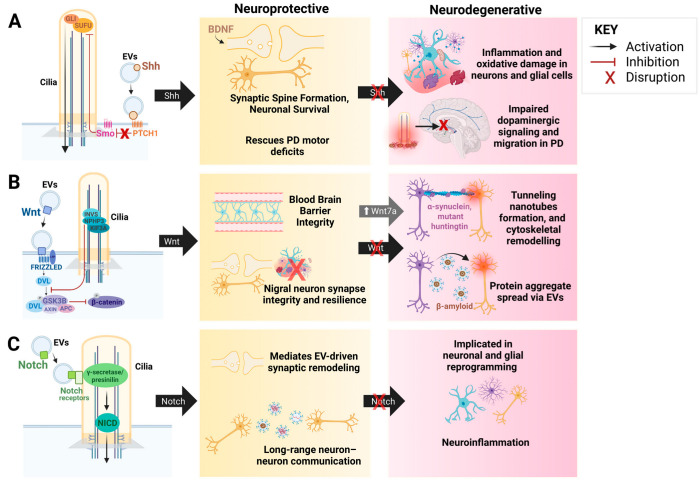
**The Interplay Between EVs, TNTs, and Ciliary Signaling Pathways: Shh, Wnt, and Notch**. This figure represents the functional interactions between EVs, TNTs, and ciliary-mediated signaling pathways, demonstrating their dual roles in neuronal maintenance and neurodegenerative disorders. (**A**). Shh signaling promotes synaptic spine formation and neuronal survival via EV-associated brain-derived neurotrophic factor (BDNF), rescuing dopaminergic neuron loss and motor deficits in PD. Dysregulation of Shh signaling impairs dopaminergic signaling and migration, contributing to inflammation, oxidative stress, and neuronal degeneration. (**B**). Wnt signaling pathway maintains blood-brain barrier integrity, synaptic resilience, and nigral neuron survival. Abnormal Wnt activity, via the Wnt7a pathway, facilitates tunneling nanotube formation, cytoskeletal remodeling, and EV-mediated propagation of protein aggregates (α-syn, Aβ, mutant huntingtin), linking this pathway to the spread of neurotoxin proteins and neuroinflammation seen in NDDs. (**C**). Notch signaling mediates EV-driven synaptic remodeling and neuron-neuron communication. Dysregulated Notch signaling may contribute to glial and neuronal reprogramming and neuroinflammation during neurodegeneration. Created in BioRender. Chen, G. (2025) https://BioRender.com/3h40i7a [290].

In the context of NDDs, PC dysfunction manifests primarily in two ways: (1) a reduction in cilia frequency or (2) alterations in ciliary length. Specifically, neurons and glial cells may either lack cilia altogether or display shortened or elongated cilia. Notably, ciliary shortening has been observed in AD and PD, whereas elongation is characteristic of HD. Although the manifestations of ciliary dysfunction vary across AD, PD, and HD, a unifying mechanism emerges through the role of PC as an integrative signaling hub. In each disease, pathogenic protein accumulation has been associated with cilia dysfunction and impairments in key pathways such as Shh, Wnt-7, and Notch, tipping the balance from neuroprotection to degeneration. The loss of these coordinated signaling diminishes neural patterning, synaptic plasticity, and mitochondrial homeostasis, neuronal resilience, rendering neuronal cells more vulnerable to inflammation and toxicity [147,264,265,266,267,268]. Further, the interplay between EVs, TNTs, and ciliary-associated Shh, Wnt, and Notch pathways represents a convergent mechanism driving the neurodegeneration in NDDs (Figure 4). Precisely, the Shh signaling pathway, supported by EV-associated brain-derived neurotrophic factor (BDNF), has been shown to promote synaptic formation and dopaminergic neuron survival, whereas its disruption exacerbates oxidative stress and neuronal vulnerability [269,270,271]. Wnt signaling pathway has also been shown to maintain synaptic integrity, mitochondrial stability, and blood–brain barrier function; while its dysregulation could upregulate Wnt7a activity to which may induce TNT formation and EV-mediated propagation of neurotoxin proteins, potentially contributing to neuroinflammation and metabolic stress [272,273,274,275,276,277,278,279,280]. Notch signaling pathway may further contribute to EV-driven synaptic remodeling and neuron-glia communication, and its alteration may trigger neuronal remodeling and neuroinflammation [281,282,283]. The crosstalk between Shh, Wnt, and Notch signaling pathways further shows the complexity of neurodegeneration when these pathways are interrupted [267]. Beyond signaling disruption, ciliary dysfunction perturbs cytoskeletal organization and vesicular trafficking, potentially linking it to dysregulated TNT and EV biogenesis [284,285,286]. Thus, while functional PC integrate protective signaling and structural stability, their dysfunction amplifies neurotoxicity by dismantling defense mechanisms and promoting aberrant intercellular protein propagation (Figure 4). While the Shh and Wnt signaling are well-established in supporting neuronal survival, synaptic integrity, and mitochondrial function, the links between Wnt7a, Notch, TNTs, EVs, and ciliary dysfunction in propagating neurotoxicity remain largely speculative and require further validation. Neverthless, all three pathways (Shh, Wnt, and Notch) can simultaneously be disrupted in AD, PD, and HD, with primary cilia loss amplifying pathway dysfunction in disease-relevant neurons. For example, Shh signaling is highly cilia-dependent and is crucial for dopaminergic neuron survival, which could be more relevant to PD [146]. Wnt/Notch signaling may retain partial function without cilia and is strongly associated with AD and HD, which may contribute to synaptic remodeling and neuroinflammation [176,287,288,289].

Epidemiologically, age is one of the major risk factors in NDDs, exacerbating neurodegenerative processes by inducing structural and functional brain changes [280,281]. Key consequences include impaired autophagy and reduced resistance to oxidative stress, both of which contribute to mitochondrial dysfunction and heightened vulnerability to neurodegenerative diseases [291,292,293]. PC and autophagy can work synergistically to regulate protein clearance, with each influencing the other’s function. Autophagy contributes to ciliogenesis by degrading negative regulators (Oral-Facial-Digital Syndrome 1, Centriolar Coiled-Coil Protein 110), modulating ciliary transport (Intraflagellar Transport Protein 20), and controlling ciliary length (Kinesin Family Member 19) [294,295,296]. Conversely, cilia serve as a site for early autophagosome formation, enabling autophagy activation, as evidenced by reduced autophagic functions in PC-depleted cells [102]. Together, autophagy and cilia form a bidirectional regulatory axis that works simultaneously to organize environmental, mechanical, and metabolic signals to maintain homeostasis [297].

Emerging evidence has also demonstrated that mitochondrial stress triggers protective ciliogenesis via protein kinase B (AKT) signaling pathway-mediated autophagy and mitophagy in a human-derived neuroblastoma cell line, a response that declines with aging [268,298]. This further supports the new view that PC may act in a unique role as a cellular timekeeper, regulating the pace of neuronal aging [299]. In line with this, Aβ-mediated circadian dysregulation in AD has been shown to disturb the intrinsic rhythmicity of cilia, thereby compromising their structural integrity and signaling capacity [196,294]. Consequently, loss of proper PC regulation accelerates neuronal aging, marked by increased oxidative stress, mitochondrial disarray, and epigenetically driven senescence that limits DNA repair in vulnerable neurons [289,290,297].

Moreover, aging may also alter intercellular communication by shifting the functions of EVs and TNTs from protective to pathogenic. Senescent cells release EVs enriched in pro-inflammatory and neurotoxic cargo, while aging impairs TNT-mediated mitochondrial transfer, resulting in reduced neuronal resilience and facilitating the spread of protein aggregates in the brain [300,301].

The crosstalk between PC, TNT, and EVs forms an integrated network regulating neuronal homeostasis and intercellular communication. However, the precise mechanism of ciEVs remains poorly defined in NDDs. While studies have shown that ciEVs are selectively released and can be altered when cilia are disrupted, these findings have yet to be validated in NDD models [302]. Similarly, TNT can be formed by distinct processes, and it remains unclear whether their molecular structure or functions are identical across NDDs [303]. Moreover, TNTs are also difficult to visualize in vivo because their nanoscale diameter, fragile structure, and rapid, transient dynamics often necessitate highly skilled imaging approaches and advanced microscopy techniques [304,305,306]. Thus, most evidence on TNT regulation in NDDs comes from in vitro studies, limiting their in vivo applicability. Notably, TNT-like structures have been observed in astrocytic tau propagation (AD models), heart development, and tumors; however, these findings are sparse, context-specific, and require advanced imaging [307,308,309,310]. Further, the reproducibility of these studies is thought to be inconsistent, raising questions about their prevalence, stability, and physiological significance in vivo [311,312,313,314]. However, diagnostic and therapeutic innovation is rapidly advancing through the use of EVs as biomarkers and delivery systems. In AD, PD, and HD, EV-based assays targeting Aβ, α-syn, LRRK2, and mutant huntingtin show promise as noninvasive diagnostic tools. Therapeutically, stem cell-derived EVs exhibit neuroprotective, anti-inflammatory, and mitochondrial-restorative effects, while exosome-mediated miRNA delivery offers experimental potential. However, challenges in yield, scalability, and target specificity remain barriers to clinical translation. Thus, future advancement approaches that incorporate integrated proteomic, transcriptomic, lipidomic, and single-EV sequencing are likely to reveal more selective and disease-specific EV signatures across NDDs. This multi-modal profiling will help differentiate cell-type-specific vesicle populations, identify post-translationally modified cargo, and uncover subtle molecular changes associated with disease progression. In parallel, dissecting the crosstalk among PC, EVs, and TNTs may illuminate how these intercellular communication pathways converge to regulate neuronal homeostasis.

## 7. Conclusions

The interplay between ciliopathies and EVs suggests a broader framework for understanding neurodegenerative diseases. Regardless of an initial age-related, genetic, and/or proteinaceous insult, a dual-hit model, where ciliary dysfunction both drives and amplifies pathogenic propagation, may explain both the early onset of genetically driven diseases, like in HD, and the progressive deterioration in aging-related diseases, such as in AD and PD. Earlier detection could enable more timely and effective interventions, and the identification of disease-specific biomarkers within exosomes may greatly improve diagnostic accuracy. Therapeutic strategies targeting the cilia, tunneling nanotubes, and exosome pathway, by stabilizing cilia structures, regulating vesicle transport, or inhibiting the spread of toxic proteins, may offer a promising approach to slow or halt the progression of neurodegenerative diseases.

## Figures and Tables

**Table 1:** A summary of current Clinical Trials and EVs-Based Diagnostic and therapeutic applications in AD, PD, and HD.

Disease	Clinical Trial	Biomarker	Source of EV	Use	Status	ID
**AD**	Ectosomes, New Biomarkers of Tau Pathology? (ECTAUSOME)	Tau and H3K9m3	CSF	Diagnostic	Completed	NCT03381482
	Saliva and Extracellular Vesicles for Neurodegenerative Diseases (MINERVA)	Aβ1-42	Saliva- Derived EVs	Diagnostic	Recruiting	NCT06869135
	Longitudinal Innate Immunity and Aging Study (LIIA)	Exosomal Innate Immune Markers	CSF, Plasma	Prognosis	Active, not recruiting	NCT03944603
	A Multimodal Approach for Clinical Diagnosis and Treatment of Primary Progressive Aphasia (MAINSTREAM)	Size and Concentration of EVs, Neurogranin, BDNF, GFAP, NFL	Plasma	Prognosis	Recruiting	NCT05730023
	Intermittent Calorie Restriction, Insulin Resistance, and Biomarkers of Brain Function	Phosphorylated Serine312-insulin Receptor Substrate-1, P-pan-Tyrosine-IRS-1 (pY-IRS-1), Akt, Tau	Neuron-Derived EVs in Plasma and CSF	Prognostic	Completed	NCT02460783
	HUC-MSC-sEV-001 Nasal Drops for Neurodegenerative Diseases	None	Adult umbilical cord-derived mesenchymal stem cell	Therapeutic	Not yet recruiting	NCT06607900
**PD**	Saliva and Extracellular Vesicles for Neurodegenerative Diseases (MINERVA)	Asyn, MCI, and NfL for all other neurological group	Saliva- Derived EVs	Diagnostic	Recruiting	NCT06869135
	Saliva and Extracellular Vesicles for PD (RaSPiD)	Salivary Raman fingerprint of PD and atypical parkinsonism	Saliva	Diagnostic	Completed	NCT05320250
	Exploring the Olfactory Mucosa, Blood and Urine for the Identification of Early Biomarkers of PD, Atypical Parkinsonisms, and Neurocognitive Disorders Due to Lewy Body Disease (EXTRAORDINARY)	Misfolded α-syn	Olfactory Mucus, and Urine-Derived EVs	Diagnostic	Recruiting	NCT06846658
	LRRK2 and Other Novel Exosome Proteins in PD	Multiple Unspecified	Urine	Diagnostic	Completed	NCT01860118
	FoxBioNet: ECV (Extracellular Vesicle)-004	LRRK2	CSF	Diagnostic	Completed	NCT04603326
	Fox BioNet Project: ECV-003	LRRK2, p1292 LRRK2, Rabs and pRabs	CSF	Diagnostic	Completed	NCT03775447
	Effect of a Progressive Treadmill Training Protocol for PD	Raman spectra of potential EV biomarkers	Blood	Prognostic	Recruiting	NCT05902065
	HUC-MSC-sEV-001 Nasal Drops for Neurodegenerative Diseases	None	Adult umbilical cord-derived mesenchymal stem cell	Therapeutic	Not Yet Recruiting	NCT06607900
	Safety of Cultured Allogeneic Adult Umbilical Cord-Derived Mesenchymal Stem Cell Exosomes for PD	None	Adult umbilical cord-derived mesenchymal stem cell	Therapeutic	Recruiting	NCT05152394
**HD**	Extracellular Vesicles for HD	Blood-based biomarker of brain HTT	Blood	Diagnostic	Recruiting	NCT06082713

## Data Availability

No new data were created or analyzed in this study. Data sharing is not applicable to this article.
